# Efficacy of topical anesthetics of lidocaine, benzocaine, and EMLA in reducing pain during inferior alveolar nerve block in schoolchildren: a randomized controlled trial

**DOI:** 10.1038/s41405-024-00275-8

**Published:** 2024-11-27

**Authors:** Mawia Karkoutly, Laila Alatassi, Lilian Azrak, Nada Bshara

**Affiliations:** Department of Pediatric Dentistry, Damascus University, Damascus, Syrian Arab Republic

**Keywords:** Paediatric dentistry, Dental anaesthesia

## Abstract

**Objectives:**

This study aimed to evaluate the efficacy of 5% EMLA cream and 8% lidocaine gel in reducing pain during inferior alveolar nerve block (IANB) compared with 20% Benzocaine in children aged 6–10 years.

**Materials and methods:**

This was a triple‐blinded, randomized, parallel‐group, active-controlled trial with three arms. 45 children were randomly assigned into 3 groups. Group 1: control group, 20% benzocaine gel (*n* = 15). Group 2: 8% lidocaine gel (*n* = 15). Group 3: 5% EMLA cream (*n* = 15). Each topical anesthetic was applied in an amount of 0.3 mL using a cotton swab for 2 min, followed by IANB administration. The following primary outcome measures were considered: pulse rate, the face, legs, activity, cry, consolability (FLACC) behavioral pain assessment scale, and the Wong-Baker FACES pain rating scale.

**Results:**

The mean score of the FLACC behavioral pain assessment scale in group 2 (2.20 ± 1.86) was slightly higher, but this result was not statistically significant *p* = (0.806). Regarding the Wong-Baker FACES pain rating scale scores, no statistically significant difference was noted between the study groups *p* = (0.593). After IANB administration, the mean pulse rate was higher in group 3 (102.40 ± 14.28). However, this difference was not statistically significant *p* = (0.351). In addition, the mean change of the pulse rate from the baseline to this time point was not statistically significant *p* = (0.638), indicating a smaller physiologic response to the painful stimulus.

**Conclusion:**

8% lidocaine gel was not superior to 20% benzocaine nor 5% EMLA in reducing pain during IANB administration.

## Introduction

Dental anxiety is a common issue among pediatric patients. In 2021, according to Grisolia et al. [1], the global prevalence of dental phobia is estimated to be 25.8% among schoolchildren. In addition, the fear of injection is the primary cause of dental treatment avoidance [2, 3]. In 2018, according to McLenon and Rogers [4], needle phobia was prevalent among 50% of children and adolescents. The fear of needles is a real issue that needs to be addressed [5]. Thus, dentists must identify effective methods of managing pain to promote better oral health outcomes [2, 3].

Many non-pharmacological behavioral guidance techniques are used to alleviate pain while performing inferior alveolar nerve block (IANB), such as various audiovisual distraction techniques, including virtual reality (VR) box [6, 7]. However, those techniques were not widely acceptable among Syrian dentists [8]. According to Cunningham et al. [9], a VR box increases anxiety during needle insertion as it blocks the field of vision causing loss of control and leading to discomfort when pediatric patients are in the supine position. In addition, although the tell-show-do (TSD) technique is considered the standard non-pharmacological behavioral guidance method [10], according to Meshki et al. [11], the TSD technique exaggerates dental anxiety. Various techniques have been proposed to alleviate pain during injection, including warming [12], adjusting the pH [13] and the rate of infiltration of local anesthetic [14], pre-cooling the injection site [15], topical anesthetics [16], and using modern devices, such as computer-controlled anesthesia delivery system [17], DentalVibe [18], and VibraJect [19]. However, to date, no standard method has been adopted to relieve pain during injection [20].

Topical anesthetics are used to manage pain caused by various dental procedures [16]. Topical anesthetics work by reversibly blocking peripheral nerves at the administration site, and they could be effective at a depth of 2–3 mm [16]. There are various types of topical anesthetics in dentistry, which are available in spray, solution, gel, patch, and ointment forms [16]. According to Fatani et al. [21], 83% of participants state that topical anesthetics make dental injections less painful, and 82.2% are less anxious.

Lidocaine is a rapid onset amine–amide anesthetic. In addition, it is widely acceptable due to its potency and low toxicity [22]. Lidocaine gel, to date, is the gold standard topical anesthetic. The onset time of lidocaine is 2 min, and a duration of 15 minutes [16]. However, benzocaine was superior to lignocaine gel in relieving pain during IANB [23], and it was the most favorite topical anesthetic among dental practitioners [24]. In addition, although lidocaine is as potent as benzocaine on alveolar mucus, its onset is more delayed [16]. Benzocaine is an ester-based topical anesthetic agent with a rapid onset time of 1 min and a duration of 10 min, and it penetrates the mucosa to a depth of 2 mm [16]. However, the main drawback of benzocaine is causing a rare yet life-threatening condition known as methemoglobinemia [25]. In addition, 8% lidocaine gel was superior to 2% lidocaine gel in topical ocular anesthesia during intravitreal injection, and higher lidocaine concentrations do not cause toxicity [26]. 8% lidocaine spray was highly effective in reducing severe intraoral pain in patients with trigeminal neuralgia without any adverse effects [27]. However, 8% lidocaine gel effectiveness in alleviating pain during dental injections has not been extensively studied.

A eutectic mixture of local anesthetics (EMLA) is a topical cream containing a combination of 2.5% lidocaine and 2.5% prilocaine. EMLA is a potent topical anesthetic cream that belongs to the amide group of local anesthetics. It is known for its excellent pain reduction properties and is commonly used in dermatology procedures. In addition, it has been used on oral mucosa to reduce pain during minor dental treatments [28, 29]. IANB is the most painful local anesthesia block due to the deep needle insertion [30], and that makes EMLA cream our concern to test because it has a melting point lower than room temperature, causing it to become liquid oil. It enables EMLA to penetrate intact skin or mucosa to a depth of 5 mm [31]. However, its relatively slow onset time of 5 min and absorption are the main disadvantages [32]. According to Svensson et al. [33], the potency of EMLA is superior to that of 2% lidocaine after 2 min on oral mucosa. However, no study has ever compared 5% EMLA cream, 8% lidocaine gel, and 20% benzocaine gel during IANB since studies in literature only focus on topical anesthetic potency on palatine mucosa. Studies comparing various topical anesthetics during IANB administration are scarce. Hence, this study aimed to evaluate the efficacy of 5% EMLA cream and 8% lidocaine gel in reducing pain during IANB compared with 20% Benzocaine in children aged 6–10 years. The null hypothesis is that 20% Benzocaine would not outperform 5% EMLA cream and 8% lidocaine gel in reducing pain during IANB.

## Materials and methods

### Study design and ethics

This was a triple‐blinded, randomized, parallel‐group, active-controlled trial with three arms. It was conducted from June 2023 to September 2023 at the Department of Pediatric Dentistry, Damascus University. Ethical approval was obtained from the ethical committee at Damascus University (N3905). It was performed by the CONSORT statement [34] and Declaration of Helsinki 2013 [35]. Written informed consent was obtained from patients’ legal guardians before enrollment. The trial was registered at the ISRCTN registry (ISRCTN11021678) on 07/11/2023.

### Recruitment and eligibility criteria

#### Inclusion criteria

1. Children aged 6–10 years.
2. Healthy children.
3. Children with no previous dental experience.
4. Children requiring IANB for non-urgent dental treatment.

#### Exclusion criteria

1. Children are allergic to the anesthetic agents used.
2. Children with dental abscesses and/or fascial space infections.
3. Special health care needs children [36].

**Fig. 1:**
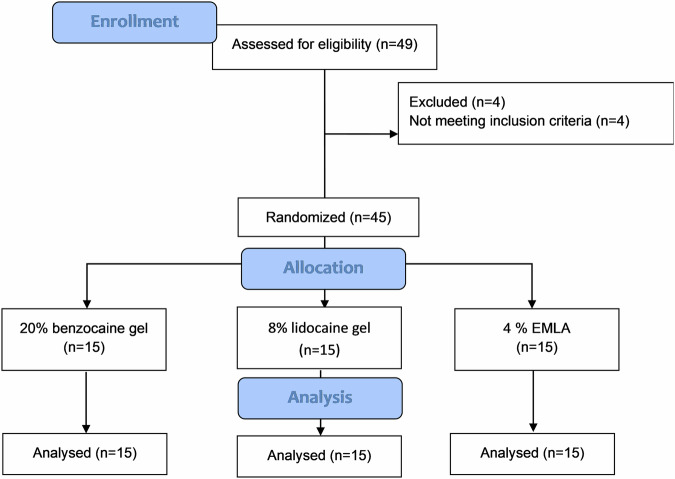
CONSORT flow diagram. CONSORT flow digram for included participants in each group.

The CONSORT flow diagram is presented in Fig. 1. 49 children were assessed for eligibility, and 4 were excluded. 45 children were randomly assigned by an experienced pediatric dentist into 3 groups according to the topical anesthetic used:

Group 1: control group, 20% benzocaine gel (Iolite, Dharma Research) (*n* = 15).

Group 2: 8% lidocaine gel (KAB Max, Kab Pharma) (*n* = 15).

Group 3: 5% EMLA cream (COSMOCAINE Plus, Al-Fares Pharma) (*n* = 15).

### Sample size calculation

The sample size was calculated using the G. Power 3.1.9 software (Heinrich‐Hein‐Universität‐Düsseldorf; http://www.gpow-er.hhu.de/). Effect size *f* = 0.4797282/α err prob = 0.05/Power (1‐β err prob) = 0.80/Number of groups = 3/Total sample size = 45. The alpha level was chosen to be 0.05 because it is the most common standard cutoff used in medical research, which means that the level of uncertainty about the study outcomes is 5%. The majority of researchers are willing to accept [37]. The power was set at 80% because most clinical trials adjust the power at 80%, which means that one in five times the statistical statistic will miss a significant difference [38]. The effect size was determined based on a preliminary study since it was calculated by dividing the mean difference of the two populations by their standard deviation [39].

### Randomization

Patients were randomized using the randomization online software https://www.randomizer.org/ by creating a randomization list for the trial. A simple randomization method was applied to randomly allocate patients into 3 groups in a ratio of 1:1:1 [40].

### Blinding

This was a triple-blinded trial where patients, outcome assessors, and data analysts were blinded to which experimental arms patients were allocated. The patients and the outcome assessors were blinded by not knowing the aim of the study and to which groups they were randomized. The data analysts were blinded by terming the groups with anonymous labels such as A, B, and C [41]. Blinding data analysts helps reduce bias and improves the credibility of the findings. Moreover, blinding as many people as feasible helps minimize bias in randomized controlled trials. It was not possible to blind the providers due to the differences in the physical properties of the materials used. A method of concealing allocation prevents interference with the randomization process, whereas blinding hides the results of the randomization. However, allocation concealment can also be referred to as “randomization blinding”.

### Intervention and primary outcome measures

The participants were randomly assigned into 3 groups. The first group received 20% benzocaine gel (control group). The second group received 8% lidocaine gel. The third group received 5% EMLA cream. Each topical anesthetic was applied in an amount of 0.3 mL [16] using a cotton swab for 2 min [42] at the site of IANB administration after drying the mucosa, and caution was taken not to cause the inhomogeneous spreading of the substance on the skin of all participants by using a small tip cotton swab. A conventional IANB was performed using a dental carpule syringe (Dental carpule syringe, Dental Laboratorio) and a 27-gauge x ¾ inch needle (Disposable Dental Needles, J Morita). The needle was inserted between the pterygomandibular raphe and the coronoid notch then aspiration was performed, and 1.8 mL of 2% lidocaine with epinephrine 1:80,000 solution (2% Lidocaine HCL Injection, Huons Co., Ltd, Seongnam) was deposited [43]. The maximum dose for a patient is calculated by multiplying the patient’s weight by the specific maximum dose per kilogram recommended for the local anesthetic being used by the dentist [16]. The following primary outcome measures were considered and evaluated by two blinded outcome assessors:

### Pulse rate assessment

Participants’ pulse rate was recorded using a finger pulse oximeter (Alpha, Prolinx GmbH) at two time points: (1) at the baseline, before IANB administration. (2) Immediately after IANB administration [44]. Pulse rate is a physiological indicator of dental pain and anxiety in pediatric patients, which is validated according to many studies [45, 46].

### Behavioral pain assessment scale

The face, legs, activity, cry, consolability (FLACC) behavioral pain assessment scale was recorded during IANB administration [47]. FLACC was designed to objectively evaluate pain in pediatric patients since it targets a population that lacks congestive and communication skills to verbalize pain [48].

### Pain rating scale

The Wong-Baker FACES pain rating scale was used to gauge the pain experienced immediately after IANB administration. Children were presented with a range of faces on the scale and asked to select the one that accurately represented their pain level during the procedure [49]. The Wong-Baker FACES pain rating scale was used to measure self-reported pain because it is easily perceived by pediatric patients as it contains facial expression illustrations [50].

Outcome assessors were calibrated by assigning the average of the scores given by the assessors who were evaluating the children. Cohen’s Kappa coefficient values of intra-examiner and inter-examiner reliability were >0.8

### Statistical analysis

Statistical analysis was carried out using the IBM SPSS software version 24 (IBM Corp.). Kolmogorov–Smirnov test was used to test the normality of data [51], and the Kruskal–Wallis test was applied to test the differences between study groups. Kruskal–Wallis test was applied because the data was not normally distributed between more than two groups of independent variables [52]. The significance level was set at *p* < 0.05.

## Results

A total of 49 children were assessed for eligibility, and 4 were excluded. 45 children were randomly allocated into 3 groups (Fig. 1). More than half of the participants were male (*n* = 26, 57.80%). The mean age was 7.71 years (standard deviation [SD] 1.18; range 6–10 years).

**Fig. 2:**
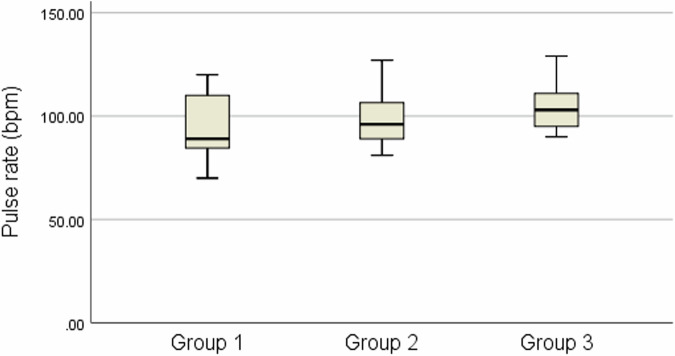
Box plots of the pulse rate after IANB administration showing median, interquartile range, minimum, and maximum.

The pulse rate assessment of the study participants is listed in Table 1. No statistically significant difference was noted between the pulse rates at the baseline *p* = (0.243), suggesting that the subjects were homogenous in terms of dental anxiety at the baseline. After IANB administration, the mean pulse rate was higher in group 3 (102.40 ± 14.28) compared with the other groups. However, this difference was not statistically significant *p* = (0.351) (Fig. 2). In addition, the mean change of the pulse rate from the baseline to this time point was not statistically significant *p* = (0.638), indicating a smaller physiologic response to the painful stimulus.

**Table 1:** Baseline demographic and clinical characteristics for each group.

Groups	*n*	Male		Female		Age	
		*n*	%	*n*	%	Mean	SD
20% benzocaine gel	15	9	60.00	6	40.00	8.06	1.16
8% lidocaine gel	15	5	33.33	10	66.67	7.73	1.43
5% EMLA cream	15	12	80.00	3	20.00	7.33	0.89
Total	45	26	57.80	19	42.20	7.71	1.18

The pain scores of the participants are listed in Table 2. The mean score of the FLACC behavioral pain assessment scale in group 2 (2.20 ± 1.86) was slightly higher compared with the other groups, but this result was not statistically significant *p* = (0.806). Regarding the Wong-Baker FACES pain rating scale scores, no statistically significant difference was noted between the study groups *p* = (0.593). However, the mean score of group 3 (1.87 ± 2.97) was slightly higher compared with the other groups Table 3.

**Table 2:** Comparison of the pulse rates between the groups.

Variables	20% benzocaine gel Mean ± SD	8% lidocaine gel Mean ± SD	5% EMLA cream Mean ± SD	DF	*p* Value
Pulse rate at the baseline	92.40 ± 12.33	95.07 ± 16.78	99.40 ± 9.70	2	0.243
Pulse rate after IANB administration	95.47 ± 15.68	99.13 ± 12.70	102.40 ± 14.28	2	0.351
Pulse rate difference (after IANB - baseline)	3.07 ± 16.39	4.07 ± 12.12	3.00 ± 13.04	2	0.638

**Table 3:** Comparison of the Wong-Baker FACES and FLACC scales between the groups.

Variables	20% benzocaine gel Mean ± SD	8% lidocaine gel Mean ± SD	5% EMLA cream Mean ± SD	DF	*p* Value
FLACC	2.00 ± 1.65	2.20 ± 1.86	1.87 ± 1.92	2	0.806
Wong-Baker FACES	0.67 ± 0.98	1.73 ± 2.49	1.87 ± 2.97	2	0.593

## Discussion

Dental pain is a common problem among individuals of all ages. It often leads to avoiding dental visits, which can damage oral health. One of the main factors contributing to dental pain is the discomfort during dental procedures, particularly needle injections [2, 3]. Therefore, several methods were used to relieve pain, such as distraction techniques and the application of topical anesthesia [6, 7]. Therefore, dentists must find the best topical anesthesia to alleviate pain during treatments [2, 3]. Thus, this study aimed to evaluate the efficacy of 5% EMLA cream and 8% lidocaine gel in reducing pain during IANB compared with 20% Benzocaine in children aged 6–10 years. To the best of the authors’ knowledge, no study has ever evaluated the efficacy of the previous topical anesthetics in reducing pain during IANB in pediatric patients.

This study included children aged 6–10 years because they are typically cooperative. In addition, they can express their pain levels accurately using pain scale measurements [53, 54]. Pulse rate was assessed in this study since it is a physiological indicator of anxiety. Research has shown that children undergoing dental treatment present increased pulse rates due to the fear and anxiety [44, 55]. The second scale used was the FLACC behavioral pain assessment scale, which provides an objective method for pain assessment. This scale is reliable and accurate in assessing pain in various populations, including young children [47, 56]. Wong-Baker Faces Pain Rating Scale was used to measure self-reported pain, which is favorable to the children [49, 57]. IANB is the most used effective anesthetic technique for various treatments in primary dentition, yet the most painful and unpleasant [58]. Therefore, several techniques were suggested to alleviate pain during injection [6–20]. The most frequently used among them is topical anesthetics [29].

In this study, 8% lidocaine gel was used due to its fast onset, low toxicity, and potency [26, 27]. In addition, 20% benzocaine gel was used because it is widely used and acceptable among dental practitioners [24]. However, benzocaine could cause various side effects, such as methemoglobinemia, soft tissue swelling, and allergic reactions [59]. 5% EMLA cream was used due to its potency in reducing pain during minor dental procedures and palatal needle injections [28]. Each topical anesthetic was applied for 2 min, which is similar to the Bhalla et al. [42] study.

In the current study, the mean change of the pulse rate from the baseline to post-IANB administration was not statistically significant, indicating a smaller physiologic response to the painful stimulus. Therefore, the three topical anesthetics are equally effective in reducing during IANB administration. In this study, the mean score of FLACC indicated that IANB injection was less painful with 5% EMLA, but this result was not significant. Regarding the Wong-Baker FACES pain rating scale scores, the mean score of the 5% EMLA group was slightly higher compared with the other groups. However, no statistically significant difference was noted between the study groups. This result is consistent with the one stated by Dasarraju et al. [60] study, which concluded that 20% benzocaine gel is not superior to 5% EMLA in reducing pain during palatal injection. However, each topical anesthetic was applied for 1 min in Dasarraju et al. [60] study. In addition, Patil et al. [61] suggested that EMLA cream is equally effective to 2% lidocaine gel in reducing pain during needle insertion. Furthermore, according to Kotian et al. [62], 5% lidocaine gel and 20% benzocaine gel are equally effective in reducing pain during IANB administration.

The result of the current study was in contrast with Nair et al. [23] findings, which concluded that 20% benzocaine is more effective than 2% lidocaine in reducing pain during IANB administration. However, the lidocaine concentration that was used in the current study is higher than the one used in the Nair et al. study. In addition, Milani et al. [63] suggested that 5% EMLA cream is superior to 20% benzocaine gel in maxillary infiltration injection for canine teeth. Moreover, according to Abu Al-Melh et al. [64], 5% EMLA is highly effective in alleviating pain during palatine injection compared to 20% benzocaine. Similarly, according to Maldonado-Ramírez et al. [65], a 5% EMLA patch was more effective than 20% benzocaine. The differences between the previous results could be attributed to the fact that pain is influenced by several psychological factors. In addition, the pain induced during local anesthetic administration could be confounded according to the injection rate, anesthetic solution pH, and the buffering capacity of the tissues [60].

In the current study, the administration of local anesthetic was performed slowly at approximately 1 mL/min because. According to de Souza Melo et al. [14], slow injections are associated with less pain and discomfort. However, in the current study, 2% lidocaine with epinephrine 1:80,000 solution was used that has approximately an acidic pH of 4. According to Sadananda et al. [66], causes greater pain sensation. The previous fact can justify the similar potency of the three topical anesthetics used in the current study. The injection is painful regardless of the topical anesthetic used. Thus, the null hypothesis in the current study was accepted since 20% of benzocaine did not outperform 5% of EMLA cream and 8% of lidocaine gel in reducing pain during IANB.

The strength of this study lies in its random allocation of participants into different groups. It helps minimize bias and increase the internal validity [67]. Additionally, the use of both physiological and behavioral scales provides a comprehensive assessment of the children’s responses to dental anxiety and pain [46]. However, this study has limitations. First, the narrow age range of participants may limit the generalizability of the findings [68]. Second, the current pain measurement tools can fail to report pain accurately. The Wong-Baker FACES pain rating scale is prone to self-report bias [57], and the FLACC behavioral pain assessment scale is subject to fake pain expressions [69]. It is recommended to test other topical anesthetic formulations. In addition, to assess pain using different pain measurement tools for children, such as artificial intelligence [70]. Furthermore, it could be beneficial to conduct future trials comparing conjunction approaches to pain management during dental injections.

## Conclusions

Based on our findings, applying topical anesthetic before IANB reduces pain in pediatric patients. However, 8% lidocaine gel was not superior to 20% benzocaine nor 5% EMLA in reducing pain during IANB administration.

## Data Availability

The datasets generated during and/or analyzed during the current study are available from the corresponding author on reasonable request.

## References

[1] Grisolia BM, Dos Santos AP, Dhyppolito IM, Buchanan H, Hill K, Oliveira BH. Prevalence of dental anxiety in children and adolescents globally: a systematic review with meta‐analyses. Int J Paediatr Dent. 2021;31:168–83. 33245591 10.1111/ipd.12712

[2] Wu L, Gao X. Children’s dental fear and anxiety: exploring family related factors. BMC Oral Health. 2018;18:1–0. 29866080 10.1186/s12903-018-0553-z PMC5987456

[3] Chakradhar K, Doshi D, Kulkarni S, Reddy BS, Reddy MP, Srilatha A. Correlation of dental anxiety with oral health status and treatment needs among 12-year old indian school going children. Acta Bio Medica: Atenei Parmensis. 2020;91:e2020095. 10.23750/abm.v91i4.8682 PMC7927553 33525260

[4] McLenon J, Rogers MA. The fear of needles: a systematic review and meta‐analysis. J Adv Nurs. 2019;75:30–42. 30109720 10.1111/jan.13818

[5] Dou L, Vanschaayk MM, Zhang Y, Fu X, Ji P, Yang D. The prevalence of dental anxiety and its association with pain and other variables among adult patients with irreversible pulpitis. BMC Oral Health. 2018;18:1–6. 29879974 10.1186/s12903-018-0563-x PMC5992818

[6] Al-Halabi MN, Bshara N, AlNerabieah Z. Effectiveness of audio visual distraction using virtual reality eyeglasses versus tablet device in child behavioral management during inferior alveolar nerve block. Anaesthesia, Pain & Intensive Care. 2018;22:55–61.

[7] Felemban OM, Alshamrani RM, Aljeddawi DH, Bagher SM. Effect of virtual reality distraction on pain and anxiety during infiltration anesthesia in pediatric patients: a randomized clinical trial. BMC Oral Health. 2021;21:321. 34172032 10.1186/s12903-021-01678-x PMC8234622

[8] Alsibai E, Karkoutly M, Abu Samra EG, Almonakel MH, Bshara N. The use of pediatric behavior management techniques among Syrian dentists: a cross-sectional study. J Global Oral Health, 10.25259/JGOH_11_2023.

[9] Cunningham A, McPolin O, Fallis R, Coyle C, Best P, McKenna G. A systematic review of the use of virtual reality or dental smartphone applications as interventions for management of paediatric dental anxiety. BMC Oral Health. 2021;21:244. 33962624 10.1186/s12903-021-01602-3 PMC8103574

[10] Dhar V, Gosnell E, Jayaraman J, Law C, Majstorović M, Marghalani AA, et al. Nonpharmacological behavior guidance for the pediatric dental patient. Pediatr Dent. 2023;45:385–410. 37904260

[11] Karkoutly M, Al-Halabi MN, Laflouf M, Bshara N. Effectiveness of a dental simulation game on reducing pain and anxiety during primary molars pulpotomy compared with tell-show-do technique in pediatric patients: a randomized clinical trial. BMC Oral Health. 2024;24:976. 39174937 10.1186/s12903-024-04732-6 PMC11342516

[12] Tirupathi SP, Rajasekhar S. Effect of warming local anesthesia solutions before intraoral administration in dentistry: a systematic review. J Dent Anesth Pain Med. 2020;20:187. 32934984 10.17245/jdapm.2020.20.4.187 PMC7470999

[13] Cepeda MS, Tzortzopoulou A, Thackrey M, Hudcova J, Arora Gandhi P, Schumann R. Cochrane review: adjusting the pH of lidocaine for reducing pain on injection. Evid‐Based Child Health: A Cochrane Rev J. 2012;7:149–215. 10.1002/14651858.CD006581.pub2 21154371

[14] de Souza Melo MR, Sabey MJ, Lima CJ, de Almeida Souza LM, Groppo FC. The effect of 2 injection speeds on local anesthetic discomfort during inferior alveolar nerve blocks. Anesth Prog. 2015;62:106–9. 26398126 10.2344/11-00037.1 PMC4581014

[15] Hameed NN, Sargod SS, Bhat SS, Hegde SK, Bava MM. Effectiveness of precooling the injection site using tetrafluorethane on pain perception in children. J Indian Soc Pedod Prevent Dent. 2018;36:296–300. 10.4103/JISPPD.JISPPD_222_17 30246753

[16] Lee HS. Recent advances in topical anesthesia. J Dent Anesth Pain Med. 2016;16:237. 28879311 10.17245/jdapm.2016.16.4.237 PMC5564188

[17] Attia S, Austermann T, May A, Mekhemar M, Conrad J, Knitschke M, et al. Pain perception following computer-controlled versus conventional dental anesthesia: randomized controlled trial. BMC Oral Health. 2022;22:425. 36138388 10.1186/s12903-022-02454-1 PMC9502910

[18] Joshi S, Bhate K, Kshirsagar K, Pawar V, Kakodkar P. DentalVibe reduces pain during the administration of local anesthetic injection in comparison to 2% lignocaine gel: results from a clinical study. J Dent Anesth Pain Med. 2021;21:41. 33585683 10.17245/jdapm.2021.21.1.41 PMC7871181

[19] Albouni MA, Kouchaji C, Al-Akkad M, Voborna I, Mounajjed R. Evaluation of the injection pain with the use of vibraject during local anesthesia injection for children: a randomized clinical trial. J Contemp Dent Pract. 2022;23:749–54. 36440524

[20] Sahithi V, Saikiran KV, Nunna M, Elicherla SR, Challa RR, Nuvvula S. Comparative evaluation of efficacy of external vibrating device and counterstimulation on child’s dental anxiety and pain perception during local anesthetic administration: a clinical trial. J Dent Anesth Pain Med. 2021;21:345. 34395902 10.17245/jdapm.2021.21.4.345 PMC8349674

[21] Fatani BA, Alhilal AI, Alkhamali IS, Alhizam AA, Alrumayyan SF, Kalanta R. Patient’s psychological perception of topical anesthetic in reducing dental needle pain: a descriptive study. J Nat Sci Med. 2023;6:137–41.

[22] Taylor A, McLeod G. Basic pharmacology of local anaesthetics. BJA Educ. 2020;20:34–41. 33456928 10.1016/j.bjae.2019.10.002 PMC7808030

[23] Nair M, Gurunathan D. Comparative evaluation of the efficacy of two anesthetic gels (2% lignocaine and 20% benzocaine) in reducing pain during administration of local anesthesia–a randomized controlled trial. J Anaesth Clin Pharmacol. 2019;35:65–9. 10.4103/joacp.JOACP_73_18 PMC6495618 31057243

[24] Alanazi FS, Alhazzaa MF, Alosaimi YM, Alajaji FA, Alanazi AS, Alassaf A, et al. Preference of dental practitioners toward the use of local and topical anesthetics for pediatric patients in Saudi Arabia: a cross-sectional survey. Children. 2021;8:978. 34828691 10.3390/children8110978 PMC8617856

[25] Rawla P, Raj JP. Methemoglobinemia: a rare entity caused by commonly used topical anesthetic agents, a case report. J Hematol. 2017;6:87. 32300399 10.14740/jh325w PMC7155844

[26] Shiroma HF. Safety and efficacy of various concentrations of lidocaine gel for intravitreal injections. Investig Ophthalmol Vis Sci. 2014;55:575. 10.1517/14740338.2014.947261 25171074

[27] Niki Y, Kanai A, Hoshi K, Okamoto H. Immediate analgesic effect of 8% lidocaine applied to the oral mucosa in patients with trigeminal neuralgia. Pain Med. 2014;15:826–31. 24506194 10.1111/pme.12349

[28] Daneshkazemi A, Abrisham SM, Daneshkazemi P, Davoudi A. The efficacy of eutectic mixture of local anesthetics as a topical anesthetic agent used for dental procedures: a brief review. Anesth Essays Res. 2016;10:383–7. 27746520 10.4103/0259-1162.172342 PMC5062240

[29] Afshari E, Sabbagh S, Khorakian F, Sarraf Shirazi A, Akbarzadeh Baghban A. Reducing pain and discomfort associated with rubber dam clamp placement in children and adolescents: a systematic review and meta-analysis of effectiveness. BMC Oral Health. 2023;23:398. 37328861 10.1186/s12903-023-03115-7 PMC10276393

[30] Krishna S, Selvarasu K, Kumar SP, Krishnan M. Efficacy of different techniques of the inferior alveolar nerve block for mandibular anesthesia: a comparative prospective study. Cureus. 2024;16. 10.7759/cureus.53277 PMC10905058 38435928

[31] Parker JF, Vats A, Bauer G. EMLA toxicity after application for allergy skin testing. Pediatrics. 2004;113:410–1. 14754960 10.1542/peds.113.2.410

[32] Singer AJ, Stark MJ. LET versus EMLA for pretreating lacerations: a randomized trial. Acad Emerg Med. 2001;8:223–30. 11229943 10.1111/j.1553-2712.2001.tb01297.x

[33] Svensson P, Bjerring P, Arendt-Nielsen L, Kaaber S. Hypoalgesic effect of EMLA and lidocaine gel applied on human oral mucosa: quantitative evaluation by sensory and pain thresholds to argon laser stimulation. Anesth Prog. 1992;39:4. 8507024 PMC2148723

[34] Cuschieri S. The CONSORT statement. Saudi J Anaesth. 2019;13:S27–30. 30930716 10.4103/sja.SJA_559_18 PMC6398298

[35] World Medical Association. World Medical Association Declaration of Helsinki: ethical principles for medical research involving human subjects. Jama. 2013;310:2191–4. 24141714 10.1001/jama.2013.281053

[36] Petluru H, Nirmala SV, Nuvvula S. A comparative evaluation of peppermint oil and lignocaine spray as topical anesthetic agents prior to local anesthesia in children: a randomized clinical trial. J Dent Anesth Pain Med. 2024;24:119. 38584755 10.17245/jdapm.2024.24.2.119 PMC10995539

[37] Miller J, Ulrich R. The quest for an optimal alpha. PLoS ONE. 2019;14:e0208631. 30601826 10.1371/journal.pone.0208631 PMC6314595

[38] Gupta KK, Attri JP, Singh A, Kaur H, Kaur G. Basic concepts for sample size calculation: critical step for any clinical trials! Saudi J Anaesth. 2016;10:328–31. 27375390 10.4103/1658-354X.174918 PMC4916819

[39] Fritz CO, Morris PE, Richler JJ. Effect size estimates: current use, calculations, and interpretation. J Exp Psychol: Gen. 2012;141:2. 21823805 10.1037/a0024338

[40] Suresh KP. An overview of randomization techniques: an unbiased assessment of outcome in clinical research. J Hum Reprod Sci. 2011;4:8–11. 21772732 10.4103/0974-1208.82352 PMC3136079

[41] Pitre T, Kirsh S, Jassal T, Anderson M, Padoan A, Xiang A, et al. The impact of blinding on trial results: a systematic review and meta‐analysis. Cochrane Evid Synth Methods. 2023;1:e12015. 10.1002/cesm.12015 PMC11795910 40475370

[42] Bhalla J, Meechan JG, Lawrence HP, Grad HA, Haas DA. Effect of time on clinical efficacy of topical anesthesia. Anesth Prog. 2009;56:36–41. 19642717 10.2344/0003-3006-56.2.36 PMC2699690

[43] Khalil H. A basic review on the inferior alveolar nerve block techniques. Anesth Essays Res. 2014;8:3–8. 25886095 10.4103/0259-1162.128891 PMC4173572

[44] Manepalli S, Nuvvula S, Kamatham R, Nirmala S. Comparative efficacy of a self‐report scale and physiological measures in dental anxiety of children. J Investig Clin Dent. 2014;5:301–6. 23766146 10.1111/jicd.12046

[45] Kothari S, Gurunathan D. Factors influencing anxiety levels in children undergoing dental treatment in an undergraduate clinic. J Fam Med Prim care. 2019;8:2036–41. 10.4103/jfmpc.jfmpc_229_19 PMC6618196 31334176

[46] Yon MJ, Chen KJ, Gao SS, Duangthip D, Lo EC, Chu CH. An introduction to assessing dental fear and anxiety in children. InHealthcare. 2020;8:86. 10.3390/healthcare8020086 PMC7348974 32260395

[47] Willis MH, Merkel SI, Voepel-Lewis T, Malviya S. FLACC behavioral pain assessment scale: a comparison with the child’s self-report. Pediatr Nurs. 2003;29:195. 12836995

[48] Rybojad B, Sieniawski D, Rybojad P, Samardakiewicz M, Aftyka A. Pain evaluation in the paediatric emergency department: differences in ratings by patients, parents and nurses. Int J Environ Res Public Health. 2022;19:2489. 35206676 10.3390/ijerph19042489 PMC8872586

[49] Wong DL. Pain in children: comparison of assessment scales. Pediatr Nurs. 1988;14:9–17. 3344163

[50] Wong DL, Baker CM. Wong-Baker faces pain rating scale. Pain Manag Nurs. 2012.

[51] Mishra P, Pandey CM, Singh U, Gupta A, Sahu C, Keshri A. Descriptive statistics and normality tests for statistical data. Ann Card Anaesth. 2019;22:67–72. 30648682 10.4103/aca.ACA_157_18 PMC6350423

[52] Mondal H, Mondal S, Majumder R, De R. Conduct common statistical tests online. Indian Dermatol Online J. 2022;13:539–42. 36262562 10.4103/idoj.idoj_605_21 PMC9574156

[53] Von Baeyer CL. Children’s self-report of pain intensity: what we know, where we are headed. Pain Res Manag. 2009;14:39–45. 19262915 10.1155/2009/259759 PMC2706563

[54] von Baeyer CL. Children’s self-reports of pain intensity: scale selection, limitations and interpretation. Pain Res Manag. 2006;11:157–62. 16960632 10.1155/2006/197616 PMC2539005

[55] Kilinç G, Akay A, Eden E, Sevinç N, Ellidokuz H. Evaluation of children’s dental anxiety levels at a kindergarten and at a dental clinic. Braz oral Res. 2016;30:e72. 10.1590/1807-3107BOR-2016.vol30.0072 27556551

[56] Peng T, Qu S, Du Z, Chen Z, Xiao T, Chen R. A systematic review of the measurement properties of face, Legs, activity, Cry and consolability scale for pediatric pain assessment. J Pain Res. 2023:16:1185–96. 10.2147/JPR.S397064 PMC10094406 37064956

[57] Garra G, Singer AJ, Taira BR, Chohan J, Cardoz H, Chisena E, et al. Validation of the Wong‐Baker FACES pain rating scale in pediatric emergency department patients. Acad Emerg Med. 2010;17:50–4. 20003121 10.1111/j.1553-2712.2009.00620.x

[58] Gajendragadkar K, Bhate K, Jagtap B, Santhoshkumar SN, Kshirsagar K, Magoo S. Making inferior alveolar nerve block more comfortable via computer-controlled local anesthetic delivery: a prospective clinical study. J Dent Anesth Pain Med. 2019;19:135. 31338419 10.17245/jdapm.2019.19.3.135 PMC6620535

[59] Memarpour M, Soltanimehr E, Eskandarian T. Signs and symptoms associated with primary tooth eruption: a clinical trial of nonpharmacological remedies. BMC Oral Health. 2015;15:1–8. 26215351 10.1186/s12903-015-0070-2 PMC4517507

[60] Dasarraju RK, Nirmala SV. Comparative efficacy of three topical anesthetics on 7-11-year-old children: a randomized clinical study. J Dent Anesth Pain Med. 2020;20:29. 32158957 10.17245/jdapm.2020.20.1.29 PMC7054072

[61] Patil SB, Popali DD, Bondarde PA, Khandare NS, Kothari AR, Chawla PS, et al. Comparative evaluation of the effectiveness of different pain-alleviating methods before local anesthetic administration in children of 6 to 12 years of age: a clinical study. Int J Clin Pediatr Dent. 2021;14:447. 34824494 10.5005/jp-journals-10005-1998 PMC8585909

[62] Kotian N, Mani G, Ramakrishnan M. Comparative evaluation of two different topical anesthetic agents in controlling pain during intraoral local anesthetic administration in children: a Split-mouth Triple-blinded Randomized Clinical Trial. Int J Clin Pediatr Dent. 2021;14:180. 34413587 10.5005/jp-journals-10005-1905 PMC8343671

[63] Milani AS, Zand V, Abdollahi AA, Froughreyhani M, Zakeri-Milani P, Jafarabadi MA. Effect of topical anesthesia with lidocaine-prilocaine (EMLA) cream and local pressure on pain during infiltration injection for maxillary canines: a randomized double-blind clinical trial. J Contemp Dent Pract. 2016;17:592–6. 27595728

[64] Al-Melh MA, Andersson L. Comparison of topical anesthetics (EMLA/Oraqix vs. benzocaine) on pain experienced during palatal needle injection. Oral Surg Oral Med Oral Pathol Oral Radiol Endodont. 2007;103:e16–20. 10.1016/j.tripleo.2006.11.033 17331753

[65] Maldonado-Ramírez MA, Issasi-Hernández H, Trejo-Tejeda S, Morales-Sánchez LA. Efficacy of two topical anesthetics for dental use in pediatric. Acta Pediátr de México. 2017;38:83–90.

[66] Sadananda V, MK J, Hegde MN, Shetty A, Gatti P. Comparison of buffered and non‑buffered lidocaine: pH and pain perception. World Acad Sci J. 2022;4:1–4.

[67] Lim CY, In J. Randomization in clinical studies. Korean J Anesthesiol. 2019;72:221. 30929415 10.4097/kja.19049 PMC6547231

[68] Ross PT, Bibler Zaidi NL. Limited by our limitations. Perspect Med Educ. 2019;8:261–4. 31347033 10.1007/s40037-019-00530-x PMC6684501

[69] Alkhouli M, Al-Nerabieah Z, Dashash M. Analyzing facial action units in children to differentiate genuine and fake pain during inferior alveolar nerve block: a cross-sectional study. Sci Rep. 2023;13:15564. 37730922 10.1038/s41598-023-42982-6 PMC10511437

[70] Hughes JD, Chivers P, Hoti K. The clinical suitability of an artificial intelligence–enabled pain assessment tool for use in infants: feasibility and usability evaluation study. J Med Internet Res. 2023;25:e41992. 36780223 10.2196/41992 PMC9972204

